# Overexpression of Ubiquitin-Specific Protease 2 (USP2) in the Heart Suppressed Pressure Overload-Induced Cardiac Remodeling

**DOI:** 10.1155/2020/4121750

**Published:** 2020-09-07

**Authors:** Junhui Xing, Pengcheng Li, Jin Hong, Mengyu Wang, Yuzhou Liu, Yueqiao Gao, Jianzeng Dong, Heping Gu, Ling Li

**Affiliations:** ^1^Department of Cardiology, The First Affiliated Hospital of Zhengzhou University, No. 1 Jianshe East Road, Zhengzhou, Henan 450052, China; ^2^Department of Endocrinology, The First Affiliated Hospital of Zhengzhou University, No. 1 Jianshe East Road, Zhengzhou, Henan 450052, China

## Abstract

Ubiquitin-specific protease 2 (USP2) is an important member of the deubiquitination system. GEO dataset revealed that USP2 was downregulated in the hearts under pressure overload. However, the cardiomyocyte-specific function of USP2 in the setting of pressure overload is unknown. In the current study, a mouse model of pressure overload was induced by transverse aortic constriction (TAC, 2 weeks). Overexpression of USP2 in the heart was conducted by AAV9 infection. Changes in heart histology were detected by Masson's trichrome staining and hematoxylin-eosin staining (H&E). Echocardiography was used to assess cardiac function. The size of cardiomyocytes was examined by wheat germ agglutinin (WGA) staining. Cardiac oxidative stress was detected by dihydroethidine staining. Our results showed that USP2 was downregulated in the cardiomyocytes following 2 weeks of TAC. Overexpression of cardiac USP2 preserved ventricular function following 2 weeks of TAC. Overexpression of cardiac USP2 inhibited TAC-induced cardiac remodeling, by suppressing cardiac hypertrophy, inhibiting inflammatory responses and fibrosis, and attenuating oxidative stress. Our findings reveal a previously unrecognized role of USP2 in regulating pressure overload-induced cardiac remodeling.

## 1. Introduction

Deubiquitinating enzymes (DUBs) are a class of proteins responsible for regulating the ubiquitin proteasome system [1]. DUBs normally belong to the cysteine protease or metalloprotease family, which trim polyubiquitin chains or remove ubiquitin from target proteins [1]. Alterations in DUBs are observed in many types of cancers, which are able to promote tumor cell proliferation or metastasis by stabilizing oncogenes or destabilizing tumor suppressors [2–5].

Heart failure is the leading cause of death worldwide, with current therapies only delaying disease progression. Cardiac hypertrophy is an early hallmark of heart failure, which is now recognized as a response to various stimulations, including pressure overload, ischemia, or metabolic disorders [6]. Overexpression or knockdown of ubiquitin enzymes contributed to the pathogenesis of myocardial infarction [7], pressure overload [8], or Ang II infusion-induced heart diseases [9], via promoting cardiac remodeling caused by the imbalance degradation system. However, little is known about the roles of DUBs in heart diseases. As the function of DUBs is opposite to that of ubiquitin enzymes, we hypothesized that alterations in DUBs could be observed in heart diseases and contribute to the disease progress.

Based on sequence and domain conservation, DUBs can be divided into six groups: ubiquitin-specific proteases (USPs), ovarian-tumor proteases (OTUs), Machado–Joseph disease protein domain proteases (MJDs), ubiquitin carboxy-terminal hydrolases (UCHs), monocyte chemotactic protein-induced protein (MCPIP), and JAMM/MPN domain-associated metallopeptidases (JAMMs) [10–13]. Among these DUBs, USPs are the most abundant classes with over 60 proteases [14]. From the GEO dataset, alternations in the ubiquitin-specific protease (USPs) pattern were observed in the mouse model of pressure overload, especially USP2, which promoted us to investigate the role of USP2 in the progression of pressure overload-induced cardiac remodeling. USP2 has been shown to be overexpressed in a variety of cancers, which is involved in promoting cancer cell proliferation [2, 5], metastasis [15], and drug resistance [16]. However, functions of USP2 in the heart are very limited. In the present study, we used AAV9 to overexpress USP2 in a mouse heart and investigated the role of USP2 under the condition of pressure overload. Our results provide a novel mechanism of DUBs in pressure overload-induced cardiac remodeling.

## 2. Materials and Methods

### 2.1. GEO Dataset Analysis

GSE1621, GSE48110, and GSE52796 datasets were downloaded from GEO (http://www.ncbi.nlm.nih.gov/geo/). GSE1621 were generated using GPL81 [MG_U74Av2] Affymetrix Murine Genome U74A Version 2 Array. GSE48110 were generated using GPL6246 [MoGene-1_0-st] Affymetrix Mouse Gene 1.0 ST Array. GSE52796 were generated using GPL6887 Illumina MouseWG-6 v2.0 expression beadchip. Although the three GEO data are not generated from the same platform, the gender and species of the mice (C57/BL, male, 8-12 weeks) used, and experimental conditions (70% constriction) were all the same [17–19].

### 2.2. Animals and Transverse Aortic Constriction (TAC) Operation

All procedures were approved by the Animal Use Committee of Zhengzhou University. Wild-type male mice (C57BL/6, 10-12 weeks old, 23-25 g, purchased from Charles River Laboratories, China) were induced into a pressure overload model by TAC as described [20]. A 7-silk suture was placed around the transverse aorta between the left common carotid artery and the brachiocephalic trunk and tied tight around both the aorta and a 27-gauge needle, which was then removed, producing a reproducible constriction about 70%. The sham group was only induced by chest open and close. Recombinant adeno-associated virus serotype 9 (AAV9) expressing full length of USP2 cDNA (AAV9/USP2) and a control (AAV9-Control) were generated by Hanheng Technologies Co., Ltd. (China). For AAV9 intervention assay, mice were randomly assigned into two groups: one group received AAV9-USP2 by tail injection at 1 × 10^11^ vg, and the other group received AAV9-Control at the same concentration two weeks before TAC. Two weeks after TAC surgery, mice were anesthetized with an excess of pentobarbital (100 mg/kg), and then, the hearts were removed for the following studies.

### 2.3. Echocardiography

A two-dimensional guided M-mode trace from parasternal short axis crossing the papillary muscle level using a 30 MHz L15-7io transducer was recorded at a depth of 2 cm and at a sweep speed of 100 mm/s (Vevo 1100, Visualsonics). All images acquired were stored digitally on a magnetic optical disc for off-line analysis. Stored images were used to analyze heart rate (HR), LV internal dimensions at end-diastole and end-systole (LVIDd, LVIDs), and LV anterior wall and posterior wall thickness of end-diastole and end-systole (LVAWd, LAPWd, LVAWs, and LVPWs). LV ejection fraction (EF%) and fractional shortening (FS%) were calculated. Measurements were taken from 5 consecutive cardiac cycles, and the average was used, in a bland fashion.

### 2.4. Histological Analysis

The hearts were fixed in 4% paraformaldehyde for 24 h and then embedded in paraffin blocks. Sections (5 *μ*m) were subjected to hematoxylin-eosin (H&E) and Masson's trichrome staining as described [20]. Wheat germ agglutinin (WGA, Thermo-fisher) staining was used to assess the size of cardiomyocytes. Digital images (5 fields) were randomly selected and captured using the microscope (BX51, Olympus) from each single heart sample. Positive staining was analyzed using ImageJ.

### 2.5. Dihydroethidine Staining

Frozen heart sections were stained with the dihydroethidine (1 *μ*M) for 1 h at 37°C. Red dihydroethidine fluorescence was detected by using a microscope (BX51, Olympus).

### 2.6. Immunofluorescence (IF)

IF was used to determine macrophage infiltration in the heart sections. The heart sections were first fixed with 4% paraformaldehyde for 24 h and then dehydrated in 30% of sucrose for 48 h. Briefly, nonspecific staining was blocked by using 10% goat serum. After blocking, 50 *μ*l of diluted primary antibody (1/100, CD68, Abcam) was applied in each section overnight at 4°C. The secondary antibody, goat anti-rabbit conjugated with Alex Fluor 546 (1/1000, Invitrogen), was applied for 30 min in the dark. After washing, the slides were mounted with Prolong Gold Antifade reagent (Invitrogen) and then detected by using a microscope (BX51, Olympus).

### 2.7. Immunohistochemistry (IHC)

IHC was used to determine the levels and distribution of USP2 in the heart sections. Heart samples were fixed in 4% phosphate-buffered paraformaldehyde for 24 h and then embedded in paraffin. Sections were immerged in the citrate-EDTA buffer and then heated in a microwave oven for 5 min to recover antigen. Goat serum (10%) was incubated to each section for blocking nonspecific staining. After 30 min blocking, diluted primary USP2 antibody (Proteintech) was applied onto each section for 1 h. Sections were finally visualized with DAB and then counter-stained with Myer's haematoxylin for 5 min, then dehydrated and mounted with DePex.

### 2.8. Real-Time PCR Analysis

The hearts were collected for the gene expression of BNP, collagen I, collagen III, IL-1*β*, IL-6, p22phox, MCP-1, NOX2, and NOX4 by real-time PCR. Total RNA was extracted with TRIzol and then mixed with master mix containing 0.6 *μ*l of random primer, 2 *μ*l of d NTP mix, 1.4 *μ*l of nuclease-free water. The mixed solution was incubated at 65°C for 5 min and then quickly chilled on ice. Subsequently, solution was mixed with first-strand buffer and DTT and then incubated for 2 min at 37°C. MMLVRT was added and incubated at room temperature for 10 min and followed by 37°C for 5 min and 70°C for 15 min. The cycling conditions of real-time PCR consisted an initial single cycle of 5 min at 95°C, then followed by 35 cycles of 30 sec at 95°C, 30 sec at 54°C, and 15 sec at 72°C (primers are listed in Table 1). The gene expression levels were quantified relative to the expression of GAPDH.

### 2.9. Isolation of Nuclear and Cytoplasmic Protein

The dissected heart tissues were placed in 300-400 *μ*l of lysis buffer containing 20 mM Tris (pH 7.9), 140 mM NaCl, 1.5 mM MgCl_2_, 1 mM EGTA, 1 mM EDTA, 1 mM DTT, 0.5% (*w*/*v*) NP-40, 0.5 mM PMSF, and protease inhibitor. After homogenization, samples were centrifuged at 5000 rpm for 10 min at 4°C, and then supernatant containing cytoplasmic protein was collected. The pellets were resuspended in nuclear extraction buffer that contained 50 mM Tris (pH 7.9), 60 mM KCl, 1 mM EDTA, 2 mM DTT, 1 mM PMSF, and protease inhibitor and centrifuged at 13,000 rpm for 15 min at 4°C. The resulting supernatant containing nuclear protein was collected.

### 2.10. Western Blot Analysis

Heart tissues were collected, and total protein was isolated with Radio-Immunoprecipitation Assay (RIPA) buffer. The protein lysate was separated by electrophoresis in SDS-PAGE gels and then transferred into a polyvinylidene fluoride (PVDF) membrane. Membranes were incubated with primary antibodies for USP2 (Proteintech), USP18 (Abcam), p-AKT (Proteintech), AKT (Proteintech), p-ERK1/2 (Proteintech), ERK1/2 (Proteintech), p-IKK*α*/*β* (Proteintech), IKK*α*/*β* (Abcam), and p65 (Abcam). After incubating with corresponding secondary antibodies, enhanced chemiluminescence reagent (Bio-Rad) was applied to the membrane, and then bands were visualized by a gel imaging system (FluorChem M, protein sample). GAPDH and Lamin A were used to verity cytoplasmic and nuclear loading consistency.

### 2.11. Statistical Analysis

Statistical analysis results are expressed as the mean ± standard deviation (SD). Groups were compared using an unpaired 2-tailed *t*-test or two-way ANOVA (GraphPad Prism). The values of ^∗^*P* < 0.05 and ^∗∗^*P* < 0.01 (AAV9-Control plus sham *vs.* AAV9-Control plus TAC); ^#^*P* < 0.05 and ^##^*P* < 0.01 (AAV9-Control plus TAC *vs*. AAV9-USP2 plus TAC); and ^$$^*P* < 0.01 (AAV9-Control plus sham *vs.* AAV9-USP2 plus sham) were considered statistically significant (*n* = 3-6/group).

## 3. Results

### 3.1. Cardiac USP2 Is Significantly Decreased in Response to Pressure Overload

**Figure 1:**
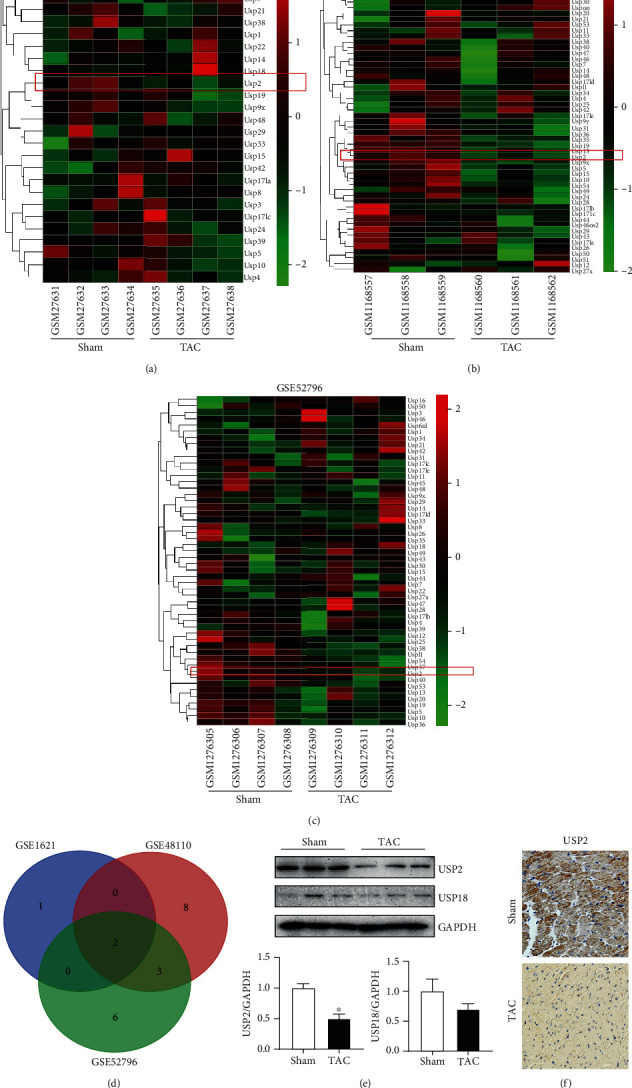
Cardiac USP2 is significantly decreased in response to pressure overload. (a–c) Hierarchical clustering and heat map analysis of TAC-related differentially expressed genes in ubiquitin-specific proteases family; (d) Venn diagrams of DGGs; (e) representative Western blot analyses of USP2 and USP18 in the heart tissues. Quantification of the relative protein levels. (f) Representative images of IHC staining of USP2 in the heart sections. ^∗^*P* < 0.05, *n* = 3/group.

We manually searched 3 GEO datasets (GSE1621, GSE48110, and GSE52796) and then conducted a comprehensive comparative analysis of USPs expression profile between the sham and TAC groups (Figures 1(a)–1(c)). After merging these datasets, decreasing USP2 and USP18 were observed in all three GEO datasets (Figure 1(d)). We further validated the content of USP2 and USP18 in both the sham and TAC groups using Western blotting. The protein levels of USP2 and USP18 were both significantly decreased, with a more profound decrease of USP2 (Figure 1(e)). IHC results further confirmed that USP2 was reduced following 2 weeks of TAC (Figure 1(f)).

### 3.2. Overexpression of USP2 in Cardiomyocytes Improved Pressure Overload-Induced Ventricular Dysfunction

**Figure 2:**
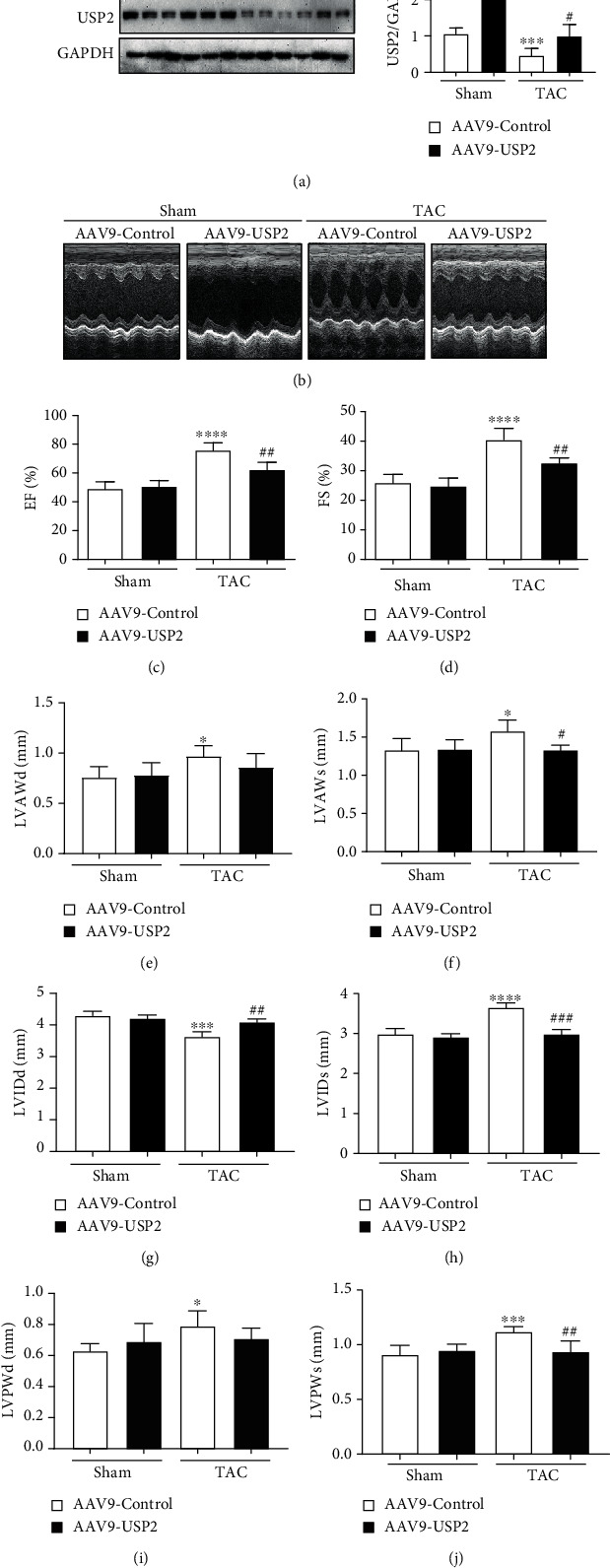
Overexpression of USP2 improves pressure overload-induced cardiac dysfunction. (a) Representative M-mode echocardiography of the left ventricular chamber and echocardiographic assessment of EF% (b) and FS% (c) from AAV-Control or AAV-USP2-treated mice following sham or TAC. Measurements of LVAWd, LVAWs, LVIDd, LVIDs, LAPWd, and LVPWs are shown in (d–i). ^∗^*P* < 0.05 and ^∗∗^ *P* < 0.01 (AAV9-Control plus TAC *vs.* AAV9-Control plus sham); ^#^*P* < 0.05 and ^##^*P* < 0.01 (AAV9-Control plus TAC *vs*. AAV9-USP2 plus TAC). *n* = 6/group.

To ensure the effect of USP2 overexpression on the cardiac contractile function, echocardiography was performed on sham or TAC-operated mice after 2 weeks. We found overexpression of USP2 markedly improved left ventricular contractile function, which was measured by EF% and FS%, as compared with the sham group after TAC operation (Figures 2(a)–2(c)). Meanwhile, LVIDd, LVIDs, LVAWd, LAPWd, LVAWs, and LVPWs were all affected in the TAC group; most of them could be preserved by overexpression of USP2 in cardiomyocytes (Figures 2(d)–2(i)).

### 3.3. Overexpression of USP2 Suppressed Cardiac Hypertrophy Induced by TAC

**Figure 3:**
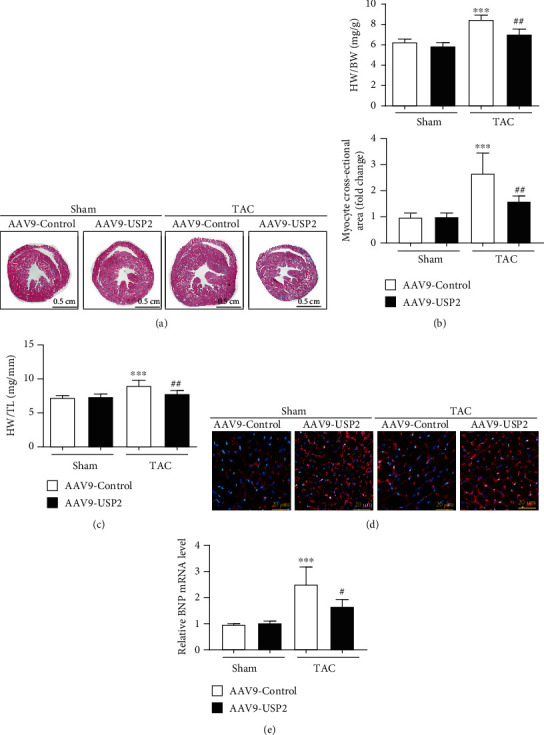
Overexpression of USP2 suppressed cardiac hypertrophy induced by pressure overload. (a) Histological analysis of heart sections from AAV9-Control or AAV9-USP2-treated mice following sham or TAC. (b and c) Heart weight to tibia length ratios (HW/TL) and body weight ratios (HW/BW) from AAV9-Control or AAV9-USP2-treated mice following sham or TAC. (d) Heart cross-sections were stained with wheat germ agglutinin (WGA). (e) Brain natriuretic peptide (BNP) mRNA levels in the hearts of AAV9-Control or AAV9-USP2-treated mice following sham or TAC. All values are presented as the relative value of the sham (AAV9-Control) value. ^∗^*P* < 0.05 and ^∗∗^*P* < 0.01 (AAV9-Control plus TAC *vs.* AAV9-Control plus sham); ^#^*P* < 0.05 and ^##^*P* < 0.01 (AAV9-Control plus TAC *vs*. AAV9-USP2 plus TAC). *n* = 6/group.

After 2 weeks of TAC, mice exhibited a markedly increase in the heart size, heart weight/tibia length (HW/TL) ratios, and heart weight/body weight (HW/BW). However, these changes were repressed in mice with cardiac USP2 overexpression (Figures 3(a)–3(c)). In addition, TAC-induced upregulation of BNP (a hypertrophic marker) and the increasing in cross-sectional areas of myocytes were also significantly inhibited in mice with cardiac USP2 overexpression after 2 weeks of TAC (Figures 3(d) and 3(e)).

### 3.4. Overexpression of Cardiac USP2 Attenuated Pressure Overload-Induced Cardiac Fibrosis and Inflammation

**Figure 4:**
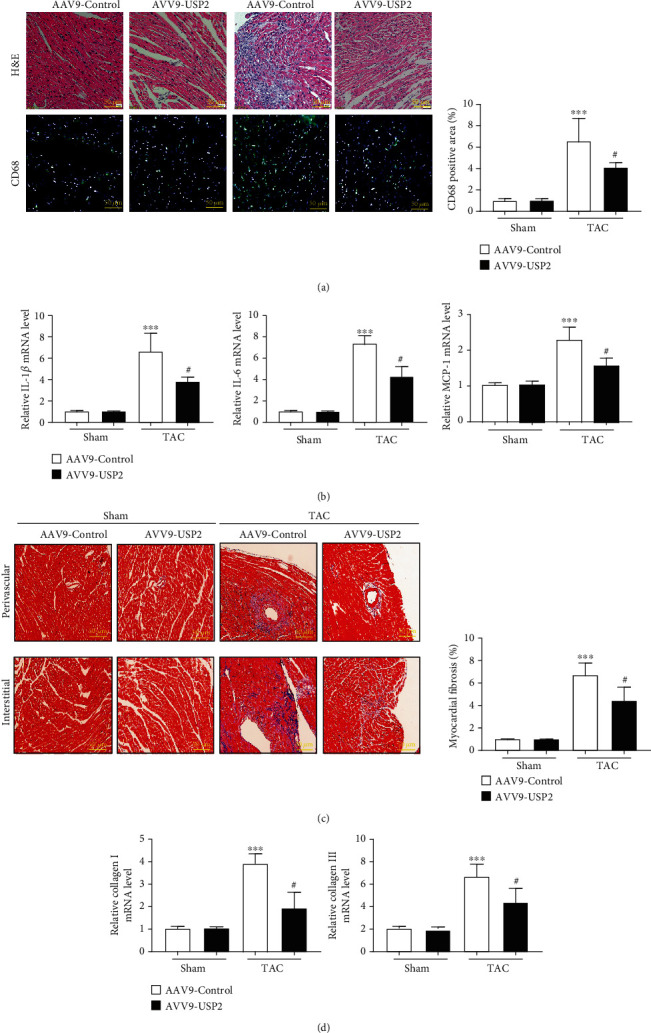
Overexpression of USP2 attenuated pressure overload-induced cardiac fibrosis and inflammation. (a) Representative images of H&E staining in the ventricular sections from AAV-Control or AAV-USP2-treated mice following sham or TAC (upper). Representative images of immunofluorescence staining in ventricular sections with CD68 antibody (lower). Quantification of CD68-positive cell areas (right panel). (b) mRNA levels of IL-1*β*, IL-6, and MCP-1 in the hearts from AAV-Control or AAV-USP2-treated mice following sham or TAC. (c) Representative images of Masson staining in the ventricular sections, showing myocardial fibrosis, and the quantification of the relative fibrotic area. (d) mRNA levels of collagen I and collagen III in heart sections. All values are presented as the relative value of the sham (AAV9-Control) value. ^∗^*P* < 0.05 and ^∗∗^*P* < 0.01 (AAV9-Control plus TAC *vs.* AAV9-Control plus sham); ^#^*P* < 0.05 and ^##^*P* < 0.01 (AAV9-Control plus TAC *vs*. AAV9-USP2 plus TAC). *n* = 6/group.

Two weeks after TAC, mice developed serve inflammatory responses. A protective effect of USP2 overexpression was detected by suppressing inflammatory cell infiltration in TAC-operated mice (Figure 4(a)). The mRNA levels of IL-1*β*, IL-6, and MCP-1 induced by TAC were significantly reduced after USP2 overexpression (Figure 4(b)). Meanwhile, TAC-induced cardiac interstitial and perivascular fibrosis were both inhibited by cardiac USP2 overexpression (Figure 4(c)). Consistently, mRNA levels of collagen I and collagen III induced by TAC were significantly reduced after USP2 overexpression (Figure 4(d)).

### 3.5. Overexpression of USP2 Reduced Cardiac Oxidative Stress in Response to Pressure Overload

**Figure 5:**
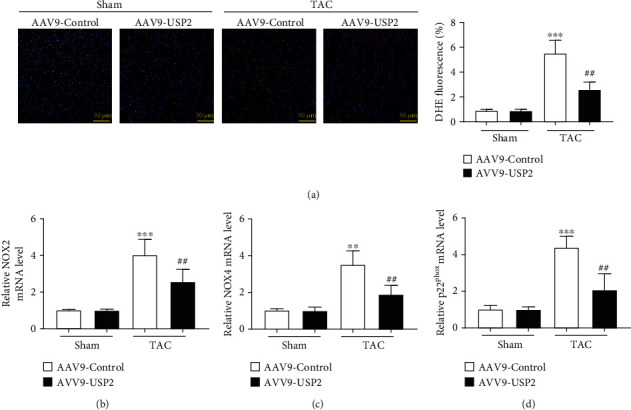
Overexpression of USP2 reduced cardiac oxidative stress in response to pressure overload. (a) Representative images of DHE in the ventricular sections (upper). Quantification of DHE fluorescence intensity (lower). (b) qPCR analysis of the mRNA expression of NOX2, NOX4, and p22^phox^ in heart tissues from AAV9-Control or AAV9-USP2-treated mice following sham or TAC. All values are presented as the relative value of the sham (AAV9-Control) value. ^∗^*P* < 0.05 and ^∗∗^*P* < 0.01 (AAV9-Control plus TAC *vs.* AAV9-Control plus sham); ^#^*P* < 0.05 and ^##^*P* < 0.01 (AAV9-Control plus TAC *vs*. AAV9-USP2 plus TAC). *n* = 6/group.

TAC significantly increased cardiac oxidative stress, as indicated by dihydroethidine staining, and this effect was abolished by USP2 overexpression (Figure 5(a)). Further, cardiac levels of NOX2, NOX4, and p22^phox^ were also significantly increased following TAC at mRNA levels, but these increasing effects were markedly reduced in the hearts with USP2 overexpression (Figures 5(b)–5(d)).

### 3.6. Overexpression of USP2 on Signaling Pathways of AKT, ERK, and NF-*κ*B

**Figure 6:**
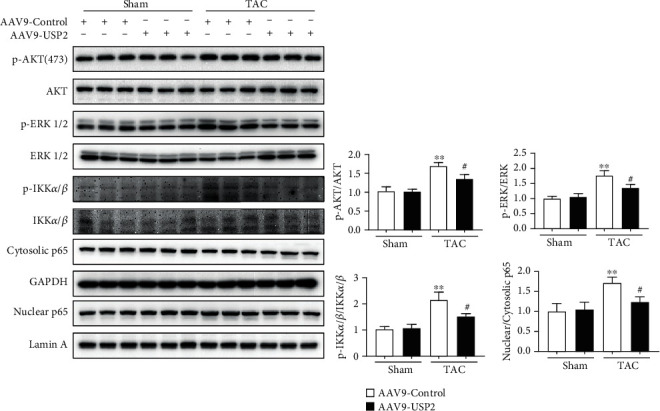
Overexpression of USP2 on signaling pathways of AKT, p-ERK1/2, and p-IKK*α*/*β*. Representative Western blot analyses of USP2, p-AKT, AKT, p-ERK1/2, ERK1/2, p-IKK*α*/*β*, IKK*α*/*β*, nuclear p65, cytosolic p65, Lamin A, and GAPDH in heart tissues from AAV9-Control or AAV9-USP2-treated mice following sham or TAC. All values are presented as the relative value of the sham (AAV9-Control) value. ^∗^*P* < 0.05 and ^∗∗^*P* < 0.01 (AAV9-Control plus TAC *vs.* AAV9-Control plus sham); ^#^*P* < 0.05 and ^##^*P* < 0.01 (AAV9-Control plus TAC *vs*. AAV9-USP2 plus TAC); ^$$^*P* < 0.01 (AAV9-Control plus sham *vs.* AAV9-USP2 plus sham). *n* = 3/group.

In order to further clarify the mechanisms of USP2 overexpression in suppression of TAC-induced cardiac remodeling, we examined the major proteins in AKT, ERK, and NF-*κ*B signaling pathways. Our results showed that the protein levels of p-AKT, p-ERK1/2, p-IKK*α*/*β*, and nuclear p65 in the mouse heart of the TAC group were significantly increased, but these increasing effects were significantly attenuated after USP2 overexpression in cardiomyocytes (Figure 6).

## 4. Discussion

In the present study, several novel findings have been made: (1) USP2 was downregulated in the heart following 2 weeks of TAC; (2) overexpression of cardiac USP2 preserved ventricular function following 2 weeks of TAC; (3) overexpression of cardiac USP2 inhibited TAC-induced cardiac remodeling, by suppressing cardiac hypertrophy, inflammatory responses, and fibrosis and attenuating oxidative stress. Our findings reveal a previously unrecognized role of USP2 in regulating pressure overload cardiac remodeling.

USP2, as an important member of the deubiquitination system, participates in tumorigenesis by regulating degradation of multiple proteins through deubiquitination, including cyclin D1, Aurora-A, cyclin A1, and Mdm2 [2–5]. Despite plenty of studies reporting the roles of USP2 in cancers, little is known about its roles in the heart. Inflammatory responses are evoked following TAC [6]. Consistently, we found TAC promoted the expression of inflammatory mediators and recruitment of macrophages to the heart. Accumulating evidence suggest that USPs are involved in immune responses and inflammation. LPS suppressed the expression of USP2 in macrophages (human myeloid HL-60 cell line) [21]. Knockdown of USP2 promoted 25 of 104 cytokines in macrophages after LPS stimulation, while overexpression of USP2 inhibited cytokine expression [21]. Upon stimulation, cardiomyocytes are able to upregulate chemokines and cytokines, which induce the first wave of inflammatory responses. We believed the levels of cardiomyocyte-derived proinflammatory mediators were inhibited by overexpression of USP2 in response to TAC, with a reduction of nuclear NF-*κ*B as an important mechanism. It has been demonstrated that overexpression of USP2 inhibited TLR/IL-1*β*-induced NF-*κ*B activation through deubiquitination of tumor necrosis factor receptor-associated factor 6 (TRAF6) in human colon cancer cells [22]. Deubiquitination of TRAF6 leads to dephosphorylation of I*κ*B and subsequently inhibition of NF-*κ*B activation [22]. However, this kind of effect is only from cancer cells, which has not been proved in cardiac cells. The reduction of NF-*κ*B pathway activation in cardiomyocytes after USP2 overexpression is due to its deubiquitinating role or by a deubiquitination-independent pathway which still requires further investigations.

The importance of ROS generation in TAC-induced heart has been well documented [23]. Depletion of USP2 in C2C12 myoblasts exhibited a higher mitochondrial ROS accumulation than control cells, associated with increased mitochondrial membrane permeability and decreased membrane potential [24]. In consistent with these findings, we found overexpression of USP2 in cardiomyocytes effectively reduced the ROS generation in the heart, but we did not measure mitochondrial functions. USP2-suppresed ROS generation is cytoplasmic or mitochondrial which still needs further investigation. Meanwhile, overexpression of USP2 also led to reduced infiltration of macrophages into the heart in response to TAC, suggesting ROS generation was likely at least in part generated from infiltrated macrophages.

Notably, we found an excessive amount of USP2 had no effect on the heart under basal conditions. Previous studies also indicated that overexpressing USP2 in cancer cell lines had no effect under the basal conditions. Upon stimulations by IL-1*β* or EGF, the activity of USP2 was elevated and further caused specific protein degradation or accumulation [22, 25, 26]. We speculated that USP2 activity may be only activated by inflammatory mediator stimulation. TAC induces severe cardiac inflammatory responses, producing a large number of inflammatory mediators. Such inflammatory mediators would be the activators of USP2.

In conclusion, here, we have for the first time uncovered a novel function of USP2 in regulating cardiac remodeling under the condition of pressure overload. The cardioprotective effects of USP2 may provide a novel therapeutic strategy for the treatment of pressure overload-induced cardiac remodeling at an early phase. Future studies, for example generating cardiac-specific USP2 knockout mice, screening target proteins, are needed to confirm the specific mechanisms of USP2 in protecting pressure overload-induced cardiac remodeling.

## Figures and Tables

**Table 1:** Sequences of primers.

Mouse BNP-forward	GAGTCCTTCGGTCTCAAGGC
Mouse BNP-reverse	ACTTCAGTGCGTTACAGCCC
Mouse collagen I-forward	CTAGCCAACCGTGCTTCTCA
Mouse collagen I-reverse	TCTCCTCATCCAGGTACGCA
Mouse collagen III-forward	TCCTGGTGGTCCTGGTACTG
Mouse collagen III-reverse	AGGAGAACCACTGTTGCCTG
Mouse IL-1*β*-forward	TGCCACCTTTTGACAGTGATG
Mouse IL-1*β*-reverse	TGATGTGCTGCTGCGAGATT
Mouse IL-6-forward	CCCCAATTTCCAATGCTCTCC
Mouse IL-6-reverse	CGCACTAGGTTTGCCGAGTA
Mouse p22-forward	GGAGCGATGTGGACAGAAGT
Mouse p22-reverse	GGCTGCCAGCAGATAGATCA
Mouse NOX2-forward	TTTGTCAAGTGCCCCAAGGT
Mouse NOX2-reverse	GGCATCTTGGAACTCCTGCT
Mouse NOX4-forward	TGTTGGGCCTAGGATTGTGT
Mouse NOX4-reverse	CAGGACTGTCCGGCACATAG
Mouse MCP-1-forward	TGAGGCTAGACCACACTCCC
Mouse MCP-1-reverse	GTCCTCAGAACCTCTGTCCG
Mouse GAPDH-forward	CCCTTAAGAGGGATGCTGCC
Mouse GAPDH-reverse	ACTGTGCCGTTGAATTTGCC

## Data Availability

The data used to support the findings of this study are available from the corresponding author upon request.
